# Synergistic peptide combinations designed to suppress SARS-CoV-2

**DOI:** 10.1016/j.heliyon.2024.e30489

**Published:** 2024-04-29

**Authors:** Tao Han, Linhong Song, Xinxin Niu, Meng Qiu, Yi Wang, Jing Wang, Xiuyan Sun, Jiali Ma, Siqi Hu, Zhichun Feng

**Affiliations:** a Department of Neonatology, Senior Department of Pediatrics, the Seventh Medical Center of Chinese PLA General Hospital, China; b Department of Pediatric Cardiac Surgery, Senior Department of Pediatrics, the Seventh Medical Center of Chinese PLA General Hospital, China; c Department of Organ Transplantation, the Third Medical Center of Chinese PLA General Hospital, China; d Senior Department of Pediatrics, the Seventh Medical Center of Chinese PLA General Hospital, China; e Institute of Pediatrics, Senior Department of Pediatrics, the Seventh Medical Center of Chinese PLA General Hospital, China; f Department of Obstetrics and Gynecology, the Seventh Medical Center of Chinese PLA General Hospital, China; g Department of Clinical Laboratory, the Seventh Medical Center of Chinese PLA General Hospital, China

**Keywords:** SARS-CoV-2, Inhibitor, Antiviral peptide, Coronavirus, COVID-19

## Abstract

The SARS-CoV-2, responsible for the COVID-19 pandemic, poses a significant threat to global healthcare. Peptide and peptide-based inhibitors, known for their safety, efficacy, and selectivity, have recently emerged as promising candidates for treating late-developing viral infections. In this study, three peptides were selected to target different stages of viral invasion, specifically ACE2 and S protein binding, as well as membrane fusion. The objective was to assess their ability to impede the entry of the SARS-CoV-2 Spike pseudotyped virus. Our findings revealed that a combination of these three peptides demonstrated enhanced antiviral effects. This outcome substantiates the feasibility of developing effective peptide combinations to combat diseases related to SARS-CoV-2. Moreover, the three-peptide combinations, designed to target multiple aspects of SARS-CoV-2 viral entry, exhibited heightened viral inhibition and broad-spectrum antiviral properties.

## 1 Introduction

Human coronaviruses (HCoVs), including HCoV-229 E, HCoV-NL63, Middle East respiratory syndrome CoV (MERS-CoV), HCoV-OC43, HCoV-HKU1, and severe acute respiratory syndrome coronavirus (SARS-CoV), can cause respiratory tract infections [1,2]. The novel coronavirus disease, COVID-19, caused by SARS-CoV-2, remains a severe threat to global healthcare [3,4]. As of April 7, 2024, it has spread globally, resulting in approximately 775.1 million cases and 7.0 million fatalities (https://covid19.who.int/).

Since the first report of the SARS-CoV-2 virus in late 2019, several variants of concern (VOC) have emerged and rapidly spread, with a worldwide distribution [5,6]. Notable variants include the alpha (B.1.1.7), beta (B.1.351), gamma (P.1), and delta (B.1.617.2) strains, along with the recently identified Omicron strain (B.1.1.529) [7]. These variants have the potential to diminish the effectiveness of current vaccinations and extend the duration of the pandemic. ACE2 (Angiotensin-converting enzyme 2), serving as the viral entry receptor, continues to facilitate the access of both the SARS-CoV-2 variants and the prototype into cells [[8], [9], [10]]. The receptor-binding domain (RBD) of the viral spike glycoprotein (S protein) [9], which is recognized by ACE2, initiates the development of a 6-helix bundle fusion core necessary for viral-host membrane adhesion [9,[11], [12], [13]]. Therefore, targeting ACE2-and RBD-binding as therapeutic candidates holds greater potential for anti-coronavirus efficacy [14,15]. Recently, Surfactin-like lipopeptides from Bacillus clausii demonstrated efficient binding of S1 and inhibition of virus entry, presenting a potential alternative for treating SARS-CoV-2 infection [16]. Studies have indicated that a peptide cocktail derived from SARS-CoV-2 can provide strong protection against viral infection [17,18]. We hypothesize that combinations of peptides targeting various stages of viral invasion (such as ACE2 and S protein binding and membrane fusion) may effectively and comprehensively prevent the spread of SARS-CoV-2 and its variants.

**Fig. 1:**
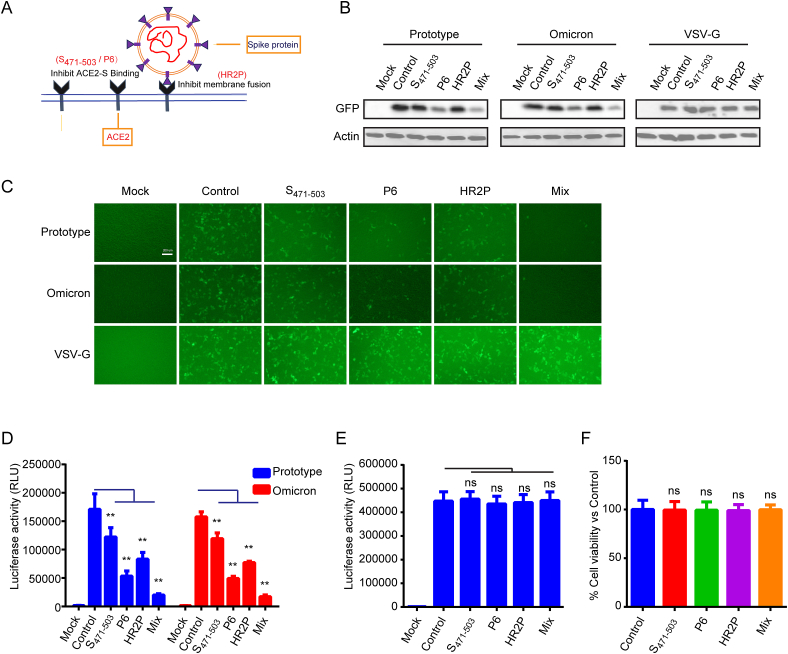
**Prevention of Spike and VSV-G pseudotypes of SARS-CoV-2 infection by the peptide.** (**A**) Mechanism of action of the peptide inhibitors targeting viral fusion and entry mediated by S protein. (**B**) Western blot analysis of lysates from SARS-CoV-2 pseudotyped virus-infected 293T hACE2 cells, confirming GFP expression. Mock indicates uninfected cells and cells infected with Spike-pseudotyped lentiviral particles serve as a control. (**C**) Fluorescence microscopy showing inhibitory activity of peptides against SARS-CoV-2 infection (green). Mock represents uninfected cells, and cells infected with Spike-pseudotyped lentiviral particles are used as a control. Representative samples from three random fields are shown (scale bars: 200 μm). (**D**) Luciferase activity of peptides vs. SARS-CoV-2 pseudotyped virus on 293T hACE2 cells. Mock indicates uninfected cells and cells infected with Spike-pseudotyped lentiviral particles are used as a control. Data represent means ± SEM from all experiments. *p < 0.05 and **p < 0.01, determined by Student's two-tailed *t*-test. (**E**) Representative graph of luciferase activity of peptides against VSV-G pseudotyped virus on 293T hACE2 cells. Mock indicates uninfected cells and cells infected with Spike-pseudotyped lentiviral particles are used as a control. Data represent means ± SEM from all experiments. *p < 0.05 and **p < 0.01, determined by Student's two-tailed *t*-test. (**F**) Cell cytotoxicity in 293T-ACE2 cells after 48 h exposure to 1 μM peptides, was evaluated using a cell proliferation assay. Data represent means ± SEM from all experiments. *p < 0.05 and **p < 0.01, determined by Student's two-tailed *t*-test. The uncropped versions of B have been provided as Supplement file. (For interpretation of the references to colour in this figure legend, the reader is referred to the Web version of this article.)

In this study, we employed three peptides targeting different stages of viral entry to assess their synergistic inhibitory effects against SARS-CoV-2 (Fig. 1A, Table 1). S_471-503_, located at the RBD of SARS-CoV, specifically inhibits the binding between the RBD and ACE2, resulting in the inhibition of SARS-CoV entry into host cells in vitro [19]. P6, composed of two discontinuous fragments (22–44 and 351–357) of ACE2 connected by glycine, robustly inhibited SARS pseudovirus infection with an IC_50_ of approximately 0.1 μM [20]. The HR2-derived peptide, HR2P, demonstrated potent inhibitory activity against S-mediated cell–cell fusion [21] (Table 1, Fig. 1A). We evaluated the antiviral efficacy of several distinct peptides against various SARS-CoV-2 VOCs. Moreover, when compared to any individual peptide, the combination of the three peptides exhibited synergistic antiviral effects with low IC_50_ values. Our results validate the concept of developing therapeutic peptide combinations against conditions related to SARS-CoV-2.

**Table 1:** Peptides used in this study.

Name	Sequence	IC_50_	Characteristic
S_471-503_ [19]	ALNCYWPLNDYGFYTTTGIGYQPYRVVVLSFEL	41.6 μM (SARS)	S-based entry inhibitor
P6 [20]	EEQAKTFLDKFNHEAEDLFYQSSGLGKGDFR	0.1 μM (SARS)	ACE2-based entry inhibitor
HR2P [21]	DISGINASVVNIQKEIDRLNEVAKNLNESLIDLQEL	0.98 μM (SARS-Cov-2)	Fusion inhibitor

## 2 Materials and methods

### 2.1 Peptide synthesis

The selected peptides were synthesized by Sangon Biotech (Shanghai, China) and purified by HPLC to > 98 % purity.

### 2.2 Cell culture

HEK 293T cells, consistently expressing recombinant human ACE2 (293T hACE2), were cultured in Dulbecco's Modified Eagle's Medium (DMEM) from Thermo Fisher. The medium was supplemented with streptomycin (100 mg/ml), penicillin (100 U/ml), and 10 % fetal bovine serum. These cells were generously provided by Dr. He Huang (Institute of Pathogen Biology, and Center for AIDS Research, Chinese Academy of Medical Sciences).

### 2.3 SARS-CoV-2 S pseudovirions development

Following a previous publication [22,23], HEK 293T cells were co-transfected to generate pseudovirions. Following the manufacturer's instructions, HEK 293T cells were co-transfected with a plasmid encoding the spike protein, a packaging construct (psPAX2), and a vector encoding the GFP and luciferase reporter (pLenti-GFP). The transfected cells produced spike-pseudotyped lentiviral particles capable of infecting cells expressing the ACE2 receptor (293T hACE2) [22,23]. Cell supernatants containing released pseudoviruses were filtered through a 0.45 μm membrane 48 h after transfection and stored at −80 °C.

### 2.4 Western blot analysis

Western blotting was performed as previously described [24]. Antibodies targeting actin (Proteintech, cat # 66009-1-Ig) and GFP (Proteintech, cat # 50430-2-AP) were employed. Cell lysates with equal protein amounts were separated by SDS-polyacrylamide gel electrophoresis (12 %) (WB1103, Beijing Biotides Biotechnology Co., Ltd.). The proteins were subsequently transferred onto nitrocellulose membranes (Whatman). The membranes were probed with the specified antibodies, followed by incubation with horseradish peroxidase-conjugated goat anti-rabbit/mouse secondary antibodies. Blots were developed using an ECL kit (1705060; Bio-Rad), and protein bands were visualized using a ChemiDoc Imaging System (Bio-Rad).

### 2.5 Immunofluorescence microscopy

293T hACE2 cells were seeded in 6-well plates prior to infection. On the following day, the test peptide was incubated for 30 min at 37 °C with equal amounts of pseudoviruses. The virus-inhibitor combination was then introduced into 293T hACE2 cells. Images were acquired 48 h post-infection at room temperature using an ECLIPSE Ts2 system (Nikon).

### 2.6 Pseudoviral transduction

The antiviral potency of each peptide was initially tested against SARS-CoV-2 spike-pseudotyped lentiviral vectors (Luc-eGFP dual reporter). These vectors were established by substituting the VSV-G fusion glycoprotein with the SARS-CoV-2 Spike protein as a surrogate viral envelope protein. A test peptide, sequentially 3-fold diluted, was incubated for 30 min at 37 °C with an equal amount of pseudovirus to determine the inhibitory activity. The virus-inhibitor combination was then introduced into 293T hACE2 cells seeded in 6-well plates. The luciferase activity of the cells was evaluated 48 h after inoculation using a Multimode Reader (Synergy Mx, BioTek, USA) after lysing the cells in a reporter lysis solution (Promega).

### 2.7 Cytotoxicity assessment of the inhibitor

The cytotoxicity assessment of the inhibitor was conducted using the CellTiter 96® AQueous One Solution from Promega. In brief, 100 μl volumes of each peptide at a concentration of 1 μM were added to cells cultured on 96-well plates at a density of 5 × 10^3^ cells/well. After 48 h of incubation at 37 °C, 20 μl of CellTiter 96® AQueous One Solution was added to each well. The optical density (OD) was measured at 490 nm using a microplate reader (Synergy HTX, BioTek).

### 2.8 Statistical analysis

All experiments were independently conducted three or more times under similar conditions. The data were presented as mean values, with the variation indicated as the standard error of the mean (SEM). Statistical significance was determined using the Student's two-tailed *t*-test, with p-values of **p < 0.01 and *p < 0.05 considered significant. IC_50_ of the inhibitors and the percentage inhibition of viral infection were calculated using GraphPad Prism software (v. 6.0, GraphPad Software Inc., USA).

## 3 Results

### 3.1 Triple-peptide cocktails confer synergistic inhibition

We selected three representative peptides, namely S_471-503_, p6, and HR2P, known to inhibit coronavirus entry (Table 1, Fig. 1A). Prior to infecting 293T hACE2 cells, a pseudotyped lentiviral vector (LV) with the SARS-CoV-2 Spike was treated with the test peptides. As controls, 293T hACE2 cells were infected with spike-pseudotyped lentiviral particles. Western blotting (Fig. 1B) and fluorescence microscopy (Fig. 1C) were employed to assess EGFP production 48 h after infection. Luciferase activity was measured in the infected 293T hACE2 cells (Fig. 1D). These results indicated a significant reduction in SARS-CoV S-mediated infection due to peptide therapy. The 3-peptide cocktail, mixed in a 1:1:1 ratio, exhibited excellent interactive inhibitory activity. Importantly, these peptides did not interfere with the pseudotyped LV-carrying vesicular stomatitis virus glycoprotein (VSV-G), a viral entry protein independent of ACE2 (Fig. 1B, C, 1E). Additionally, cell viability was assessed using a CCK8 assay 48 h after peptide treatment. As shown in Fig. 1F, these peptides demonstrated no toxicity in 293T hACE2 cells at a concentration of 1 μM.

### 3.2 IC_50_ of triple-peptide cocktails is significantly lower than that of any individual peptide

**Fig. 2:**
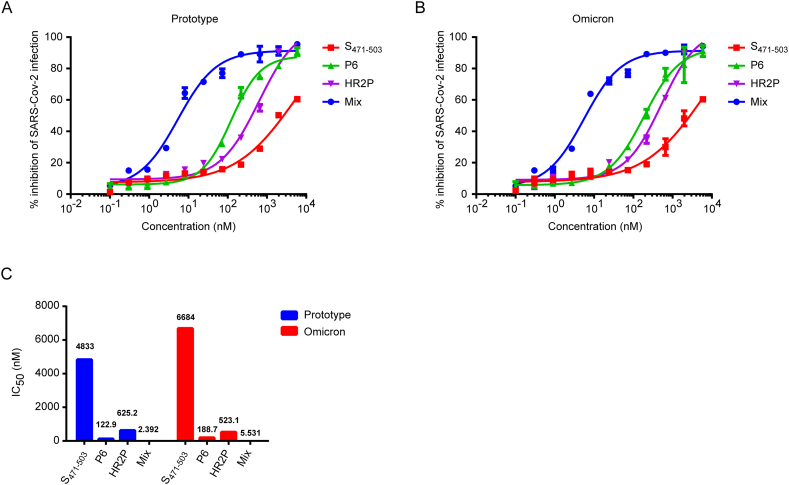
**In a dose-dependent manner, all peptides reduced SARS-CoV-2 pseudotyped viral infection.** (**A**) A single-cycle infection assay was used to test the inhibitory properties of peptides against the infections of prototype pseudoviruses on 293T hACE2 cells. Peptide dose-response curves have been established by mapping the % viral inhibition (y-axis) vs the log transformation of peptide concentration. (**B**) A single-cycle infection assay was used to determine the inhibitory properties of peptides against Omicron pseudovirus infections on 293T hACE2 cells. Mapping the % viral inhibition (y-axis) vs the log transformation of peptide concentration resulted in dose-response curves for the selected peptides. (**C**) IC_50_ of different peptides in SARS-CoV-2 pseudotyped virus.

Next, we want to detect IC_50_ of these peptides against SARS-CoV-2. Luciferase activity was assessed 48 h after introducing the peptide-pseudovirus mixture into 293T hACE2 cells (Fig. 2A and B). The IC_50_ values for S_471-503_, P6, and HR2P against the prototype pseudovirus were 4833 nM, 122.9 nM, and 625.2 nM, respectively (Fig. 2C). Correspondingly, the IC_50_ values for S_471-503_, P6, and HR2P against the Omicron pseudovirus were 6684 nM, 188.7 nM, and 523.1 nM, respectively (Fig. 2C). The 3-peptide cocktail mixed at a 1:1:1 ratio exhibited excellent interactive inhibitory activity (Fig. 1D). The IC_50_ values of the three peptides were calculated as described above. Demonstrating good synergistic antiviral activity, the three-peptide cocktail, mixed in a 1:1:1 ratio, inhibited prototype and Omicron with IC_50_ values of 2.392 and 5.531 nM, respectively (Fig. 2C). These results indicate that combining the three peptides could broaden treatment options for SARS-CoV-2 infection (Fig. 2). Furthermore, the combination effects of 2 peptides (S_471-503_+P6, P6+HR2P, S_471-503_+HR2P) in 1:1 ratio were analyzed using the combination index (CI) method and the CompuSyn software (Biosoft, CA, United States) was used for the CI calculations [25,26]. The CI model quantifies the potential interactions between drug–drug combinations into three categories: (a) synergistic effect: CI value < 1, (b) additive effect: CI = 1, and (c) antagonistic effect: CI value > 1. As shown in Table 2, three cocktails showed syneristitic effect compared to the individual components at IC_50_ concentration. In conclusion, these three peptides were validated to act synergistically to prevent the entry of SARS-CoV-2 and therapeutic intervention was likely to be the most efficient.

**Table 2:** CI values calculated by CompuSyn software.

Peptide	CI (Prototype)	CI (Omicron)
S_471-503_+P6	0.11844	0.08724
S_471-503_+HR2P	0.00888	0.01727
P6+HR2P	0.11174	0.09817

## 4 Discussion

The effectiveness of currently available drugs is under severe threat due to rapidly evolving SARS-CoV-2 variants, such as Omicron and Deltamicron, which harbor mutations in crucial epitopes targeted by RBD-specific antibodies and vaccines. Host-directed treatment (HDT) emerges as a viable strategy to develop broad-spectrum antiviral drugs addressing this challenge. HDT involves interfering with host cell factors necessary for pathogen replication or persistence [27]. It comprises two main strategies: (1) targeting host cell mechanisms, such as common components used for viral entry or replication, and (2) enhancing immune responses against infections while reducing inflammation. Promising approaches include antivirals directed at viral entry and membrane fusion processes. The SARS-CoV-2 virus infiltrates host cells through the S protein, similar to other coronaviruses. The surface S protein of SARS-CoV-2 plays a pivotal role in viral attachment to the host cell and the fusion of viral RNA within the host cell membrane [28].

The S1 and S2 subunits constitute the S protein. An RBD in the S1 subunit is responsible for attaching to ACE2 cell surface receptors [28]. The S2 component facilitates fusion between the host cell and the virus in the membrane. It includes a cytoplasmic tail, heptad repeat (HR) 1 and HR2 transmembrane domains, and an N-terminal fusion peptide (FP) [29]. Most coronavirus S proteins remain unchanged in the HR1 and HR2 domains. Antiviral drugs targeting these domains could have a broad spectrum of effects [21,[30], [31], [32], [33]]. Numerous peptides have been effectively used to impede the entry of SARS-CoV into cells [34,35]. Recently, recombinant EK1 peptide and fusion inhibitor HY3000 have been showed potent antiviral activity against SARS-CoV-2 and its variants including XBB.1.5, EG.5, EG.5.1, as well as XBB.1.16, FL.1.5.1, FY.3 and BA.2.86 [[36], [37], [38]]. In our investigation, we selected three peptides with distinct properties to target and obstruct different SARS-CoV-2 entry phases. S_471-503_ has the potential to selectively inhibit the binding of ACE2 to the RBD of SARS-CoV, thus preventing SARS-CoV infection [19]. With an IC_50_ of approximately 0.1 μM, P6, composed of two discontinuous segments of ACE2 chemically joined together by glycine, demonstrated potent antiviral activity [20]. Pan-CoV fusion inhibitor HR2P effectively inhibits SARS-mediated cell-cell fusion and SARS-CoV-2 pseudovirus infection [21].

In this study, we investigated the inhibitory potential of three peptides, namely S_471-503_, P6, and HR2P, targeting distinct stages of SARS viral invasion—specifically ACE2 and S protein binding, as well as membrane fusion. These peptides were chosen to evaluate their capacity to impede the entry of the SARS-CoV-2 Spike pseudotyped virus. Notably, when the three peptides were combined at a 1:1:1 ratio, a pronounced synergistic effect was observed (Fig. 2). S_471-503_, located at the RBD of SARS-CoV, and P6, composed of two discontinuous fragments (22–44 and 351–357) of ACE2 connected by glycine, both demonstrated the ability to specifically obstruct the binding between RBD and ACE2. Additionally, HR2P exhibited inhibitory activity against S-mediated cell–cell fusion. The combined action of these three peptides, each targeting different stages of SARS viral invasion, elucidates the mechanism underlying their synergy.

Antimicrobial peptides (AMPs) are small peptides with efficacy against a wide spectrum of microorganisms, including bacteria, fungi, parasites, and viruses [[39], [40], [41], [42]]. Combining antimicrobial peptides (AMPs) with traditional antibiotics presents a potential alternative to enhance antibacterial efficiency, concurrently reducing bacterial drug resistance [43]. The AMP database (Data Repository of Antimicrobial Peptides [DRAMP], http://dramp.cpu-bioinfor.org/) has reported over 100 AMPs exhibiting anti-SARS-CoV-2 activity [44]. Both viral and host factors serve as potential targets for antiviral therapy [45]. Mechanistically, peptides hinder SARS-CoV-2 entry into host cells, primarily by impeding its binding to host cell receptors or concealing viral peptides crucial for a successful infection. Recently, nanoformulation approaches have emerged as promising strategies to enhance distribution and stability against viral infections [46]. In comparison to biologics, therapeutic peptides exhibit high affinity and specificity by binding to cell surface receptors and initiating intracellular effects. Moreover, they boast lower manufacturing expenses and reduced immunogenicity [47].

## 5 Conclusion

The present study reveals that the combined use of three peptides exhibits enhanced antiviral effects. Our findings underscore the significance of developing therapeutic peptide combinations to combat SARS-CoV-2-related disorders. In this investigation, peptides targeting both the virus (S protein) and host factors (ACE2) effectively hinder protein-protein interactions between the S protein and ACE2, as well as cell-cell fusion. The synergistic antiviral activity of the three-peptide combinations was demonstrated by their low IC_50_ values. These peptide combinations, targeting various SARS-CoV-2 viral entry points, offer more robust virus inhibition and broad-spectrum antiviral properties.

## 6 Limitations of the study

The present study focused on in vitro viral entry assays utilizing cellular models. However, we did not examine the ratios of these three peptides. Further investigations are necessary to establish the optimal peptide dosage.

## Funding

This study was supported by the Family Planning Special Project of the People's Liberation Army (grant number 20JSZ16), and the 10.13039/501100001809National Natural Science Foundation of China (grant number 82341095).

## Data availability statement

The data are included in the article/supp. Materials/referenced in this article. The related data are available from the corresponding authors upon request. No restrictions were imposed on data security.

## CRediT authorship contribution statement

**Tao Han:** Writing – original draft, Supervision, Formal analysis, Conceptualization. **Linhong Song:** Writing – original draft, Formal analysis, Conceptualization. **Xinxin Niu:** Formal analysis. **Meng Qiu:** Resources. **Yi Wang:** Writing – original draft, Software. **Jing Wang:** Resources. **Xiuyan Sun:** Resources. **Jiali Ma:** Resources. **Siqi Hu:** Writing – original draft, Supervision, Formal analysis, Conceptualization. **Zhichun Feng:** Supervision, Resources, Conceptualization.

## Declaration of competing interest

The authors declare that they have no known competing financial interests or personal relationships that could have appeared to influence the work reported in this paper.
