# Supplementary figures and images for: Synergistic peptide combinations designed to suppress SARS-CoV-2
Source: Heliyon. 2024 Apr 29;10(9):e30489. doi: 10.1016/j.heliyon.2024.e30489 (PMC11079089; doi:10.1016/j.heliyon.2024.e30489)

Original images of Figure 1B

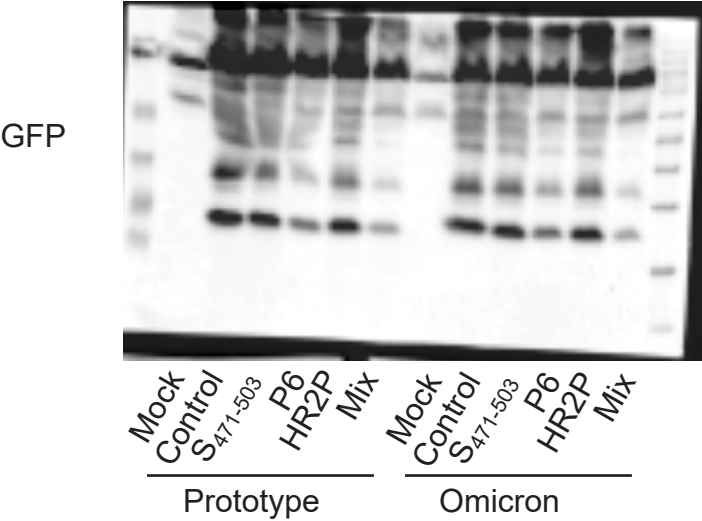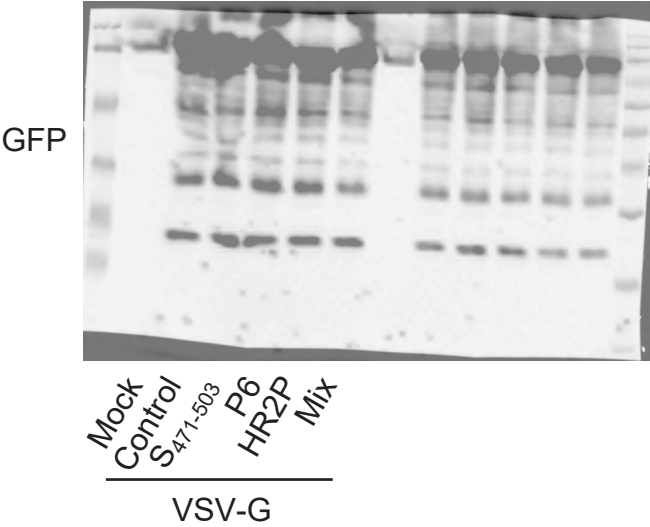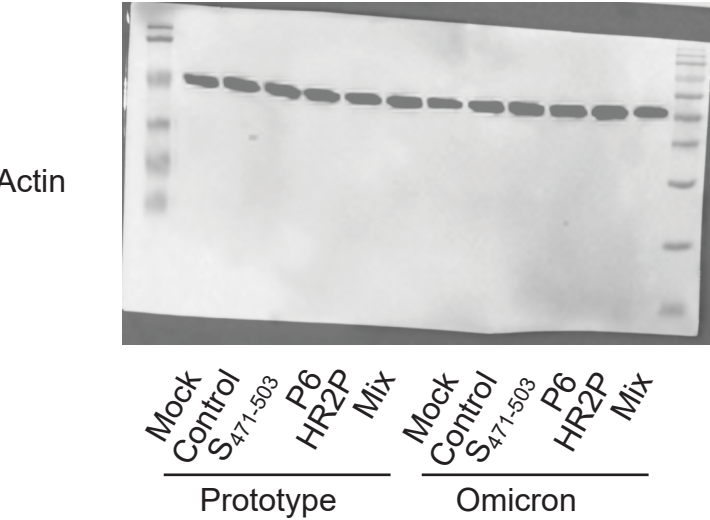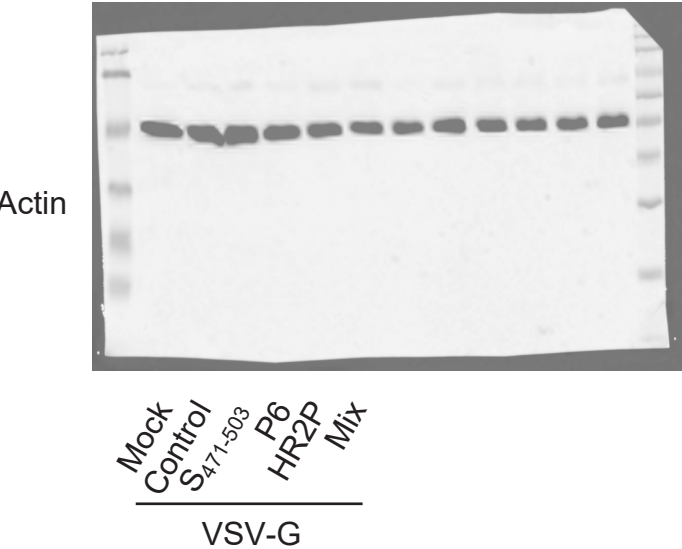

Supplement: Multimedia component 1 [file mmc1.pdf]
